# Overlapping obstructive sleep apnea and chronic obstructive pulmonary disease in patients undergoing percutaneous coronary intervention

**DOI:** 10.1007/s00392-023-02226-z

**Published:** 2023-05-22

**Authors:** C. Pizarro, L. Biener, G. Nickenig, D. Skowasch

**Affiliations:** Department of Internal Medicine II, Cardiology, Pneumology, Angiology, University Hospital Bonn, Venusberg-Campus 1, 53127 Bonn, Germany

Sirs,

Obstructive sleep apnea (OSA), characterized by repetitive collapse of upper airways during sleep, and chronic obstructive pulmonary disease (COPD), defined by persistent, usually progressive airflow limitation, are both common disorders that entail major morbidity and mortality risks [1]. Their coexistence is denominated overlap syndrome (OS). According to current estimates, the prevalence of COPD and OSA is at least 10%, respectively, but whether each disorder predisposes to a higher incidence of the other remains undefined [2]. OS carries additional prognostic implications particularly related to cardiovascular mortality. The interrelationship between atherogenesis, COPD and OSA is presumably inflammation-mediated by shared systemic inflammation pathways. Repetitive hypoxemia actuates inflammatory processes, induces endothelial dysfunction and promotes atherogenesis.

In this study, we sought to determine the prevalence of COPD, OSA and OS in patients undergoing percutaneous coronary intervention due to acute coronary syndrome, stable angina or symptom-free coronary artery disease (CAD).

Patients prospectively attending the Heart Centre Bonn (University Hospital Bonn, Germany) for inpatient coronary angiography were included in this cohort trial. Exclusion criteria for study participation comprised previously diagnosed COPD or sleep-disordered breathing (SDB). All patients underwent pulmonary function testing that encompassed spirometry, body plethysmography and determination of diffusion capacity for carbon monoxide (DL_CO_). Diagnoses of COPD were established by a post-bronchodilator Tiffeneau index < 0.7 [3]. Additional capillary blood gas analysis was performed. Nocturnal SDB screening was conducted by use of the portable SOMNOcheck micro^®^ device (Weinmann Medical Technology, Hamburg, Germany). It relies on a two-channel algorithm that permits assessment of respiratory flow, sleep fragmentation status and differentiation between obstructive and central apnoeic events [4]. SDB results were obtained by automatic data processing. At admission, prior to coronary angiography, blood samples were acquired. Written informed consent was obtained from each participant for this ethically approved study (Medical Ethics Committee of the University Hospital Bonn, Germany). All statistical analyses were performed using SPSS statistics version 26.0 (IBM, Armonk, NY, USA). Differences in categorical variables were analyzed by Fisher´s exact test. Univariate analysis of variance was used for comparison of continuous data between groups. If the global test was significant, Bonferroni correction was applied for post hoc analysis. Correlation was assessed by use of Pearson’s correlation coefficient. Statistical significance was assumed when the null hypothesis could be rejected at *p* < 0.05.

A total of 57 patients completed assessments. Overall, patients were late middle-aged (66.6 ± 10.4 years), predominantly male (89.5%) and slightly overweight (body mass index, BMI, 28.5 ± 5.5 kg/m^2^). With regard to the indication for coronary angiography, the majority presented acute coronary syndrome (*n* = 29, 50.9%), 16 patients were symptom free and received coronary angiography for completion of myocardial revascularization, the remaining 12 patients (21.1%) offered stable angina.

Pulmonary function testing revealed a prevalence of previously undiagnosed COPD of 19.3% (11/57 patients). Within this COPD cohort, forced expiratory volume in one second (FEV_1_) accounted for 1.8 ± 0.4 l in absolute terms and 64.6 ± 11.4% of the predicted value. Remarkably, 9 out of 11 COPD patients presented overlapping OSA. OSA in the absence of COPD was identified in 26 out of 57 patients (45.6%), whilst 11 patients (19.3%) exhibited neither SDB nor obstructive airflow limitation. Further 11 patients (19.3%) presented primarily central sleep apnea (CSA).

Table 1 summarizes intergroup differences as a function of pulmonary function and SDB testing results. No differences were observed in terms of demographics or cardiac measurements. Except for serum creatinine concentration that was highest amongst OS patients (*p* = 0.03) and thrombocyte count that was highest amongst patients without SDB (*p* = 0.02), biochemical parameter did not vary over groups. With regard to pulmonary function measurements, we ascertained significant differences in parameters indicating obstructive ventilatory limitation (FEV_1_, Tiffeneau index) by comparison of the OS cohort with both the OSA group (FEV_1_ absolute: *p* = 0.001; FEV_1_% predicted: *p* < 0.001; Tiffeneau index: *p* < 0.001) and patients without SDB (FEV_1_ absolute: *p* = 0.03; FEV_1_% predicted: *p* = 0.004; Tiffeneau index: *p* < 0.001). Beyond parameters that are directly connected to COPD and considering that residual volumes did not substantially differ between groups, forced vital capacity (FVC) % predicted was lowest among OS patients. Post hoc Bonferroni correction revealed significant differences in FVC % predicted between the OS and OSA group (*p* = 0.004). Likewise, lowest values for transfer factor were detected in OS patients and reached statistical significance when compared to both patients without SDB (*p* = 0.02) and OSA patients (*p* = 0.04). In terms of somnological measurements, the apnea–hypopnea index (AHI) and the oxygen desaturation index (ODI) significantly differed over groups (*p* = 0.004 and *p* = 0.04, respectively). These somnological results were exclusively driven by differences between the patient group without SDB, on the one hand, and the OSA and OS patient groups, on the other hand. No differences in AHI and ODI were observed by comparison of OSA with OS patients (*p* = 1.00 and *p* = 1.00, respectively).

**Table 1:** Characteristics of study population, stratified by pulmonary function and somnological testing results

	No sleep-disordered breathing (*n* = 11)	Obstructive sleep apnea (*n* = 26)	Overlap syndrome (*n* = 9)	*p* value
Demographics				
Age [years]	65.2 ± 10.4	65.8 ± 11.0	73.8 ± 5.3	0.08
Male sex	9 (81.8%)	23 (88.5%)	8 (88.9%)	0.84
BMI [kg/m^2^]	25.7 ± 4.3	28.9 ± 6.1	30.6 ± 6.5	0.16
Cardiac measurements				
LV ejection fraction [%]	58.0 ± 14.8	56.2 ± 11.8	48.7 ± 11.4	0.84
Coronary artery disease				0.79
Single-vessel disease	3 (27.3%)	5 (19.2%)	1 (11.1%)	
Two-vessel disease	4 (36.4%)	7 (26.9%)	2 (22.2%)	
Three-vessel disease	4 (36.4%)	14 (53.8%)	6 (66.7%)	
Symptomatology at admission				0.82
Symptom-free	3 (27.3%)	6 (23.1%)	2 (22.2%)	
Stable Angina	3 (27.3%)	5 (19.2%)	3 (33.3%)	
Unstable Angina	1 (9.1%)	7 (26.9%)	1 (11.1%)	
NSTEMI	2 (18.2%)	4 (15.4%)	3 (33.3%)	
STEMI	2 (18.2%)	4 (15.4%)	0 (0%)	
Biochemistry				
Cardiac troponin I [ng/ml]	1.1 ± 1.2	14.9 ± 39.4	0.06 ± 0.04	0.55
Hs-CRP [mg/l]	5.2 ± 6.2	18.8 ± 23.5	10.1 ± 7.3	0.33
Serum creatinine [mg/dl]	1.1 ± 0.4	1.2 ± 0.4	1.7 ± 0.8	**0.03**
Leukocytes [10^9^/ml]	8.4 ± 3.1	8.7 ± 3.5	7.8 ± 2.8	0.78
Neutrophils [% of leukocytes]	42.8 ± 39.7	50.0 ± 26.0	49.4 ± 43.1	0.88
Hemoglobin [g/dl]	13.7 ± 2.2	13.8 ± 1.9	12.7 ± 1.8	0.30
Thrombocytes [10^9^/ml]	333.8 ± 217.1	217.9 ± 67.9	203.8 ± 37.5	**0.02**
Pulmonary function parameters and capillary blood gas analysis				
FVC [l]	3.4 ± 1.0	3.6 ± 0.8	2.8 ± 0.7	0.06
FVC [% predicted]	85.2 ± 15.7	91.1 ± 13.0	73.6 ± 9.4	**0.006**
FEV_1_ [l]	2.8 ± 0.9	3.0 ± 0.7	1.9 ± 0.4	**0.001**
FEV_1_ [% predicted]	89.0 ± 15.2	98.3 ± 15.2	66.5 ± 11.6	** < 0.001**
FEV_1_/VC [%]	82.9 ± 6.1	83.7 ± 5.8	68.1 ± 5.0	** < 0.001**
RV [l]	2.8 ± 1.0	2.8 ± 0.7	3.4 ± 1.2	0.24
RV [% predicted]	114.2 ± 34.7	115.4 ± 29.3	127.4 ± 44.4	0.62
DL_CO_ [% predicted]	71.5 ± 14.0	67.8 ± 13.9	47.8 ± 13.4	**0.02**
DL_CO_/VA [% predicted]	88.3 ± 13.6	83.1 ± 13.2	69.8 ± 17.7	0.09
pO_2_ [mmHg]	76.5 ± 6.9	73.4 ± 8.8	65.4 ± 8.5	**0.03**
pCO_2_ [mmHg]	30.8 ± 2.7	34.1 ± 2.5	35.9 ± 3.9	**0.001**
sO_2_ [%]	96.1 ± 0.9	93.9 ± 5.9	93.2 ± 2.2	0.34
pH	7.47 ± 0.03	7.44 ± 0.04	7.45 ± 0.03	**0.04**
SDB screening				
Recording time [h]	5.8 ± 1.7	6.3 ± 1.8	6.7 ± 0.8	0.42
AHI	3.1 ± 1.4	21.3 ± 17.3	24.2 ± 18.8	**0.004**
Autonomic arousals index	19.9 ± 12.3	17.4 ± 13.5	6.7 ± 7.1	0.09
ODI [per h]	1.2 ± 0.8	7.2 ± 8.8	9.2 ± 6.9	**0.04**
Minimal saturation [%]	85.8 ± 4.7	81.4 ± 10.8	76.0 ± 10.6	0.09
Mean saturation [%]	95.9 ± 1.1	93.9 ± 3.1	92.6 ± 5.2	0.08
Nocturnal heart rate [per min]	58.5 ± 9.9	59.7 ± 6.9	64.1 ± 10.4	0.38
Epworth sleepiness scale	4.0 ± 2.8	7.0 ± 4.1	8.0 ± 5.4	0.08

Data exclude those patients in whom SDB screening revealed central sleep apnea (*n* = 11). Dare are presented as n (%) or mean ± standard deviation. *p* values are significant at < 0.05. Statistically significant differences are given in bold

*AHI* apnea–hypopnea index, *BMI* body mass index, *DL*_*CO*_ diffusion capacity of the lung for carbon monoxide, *FEV*_*1*_ forced expiratory volume in 1 s, *FVC* forced vital capacity, *Hs-CRP* high-sensitivity C-reactive protein, *LV* left ventricular, *NSTEMI* non-ST-elevation myocardial infarction, *ODI* oxygen desaturation index, *PCO*_*2*_ carbon dioxide tension, *PO*_*2*_ oxygen tension, *RV* residual volume, *SDB* sleep-disordered breathing, *sO*_*2*_ oxygen saturation, *STEMI* ST-elevation myocardial infarction, *VA* alveolar volume

When CSA patients were analyzed for intergroup differences by analogy with Table 1, the only significant difference that affected the CSA patient group resulted from comparison of the AHI and oxygen desaturation index (ODI) between CSA patients and patients without SDB (AHI: 23.5 ± 22.1/h vs. 3.1 ± 1.4/h, *p* = 0.02; ODI: 23.4 ± 26.2/h vs. 1.2 ± 0.8/h, *p* = 0.003).

Atherosclerotic conditions, primarily CAD, have repeatedly been associated with OSA in prospective and cross-sectional studies [5]. Whereas OSA shows a prevalence of 9–24% in the unselected general population [6], we presently ascertained a prevalence of 61.4% among CAD patients. Overlapping COPD was detected in 9 out of 35 OSA patients. Consequently, the frequency of OS in the entire study population was 15.8%. This frequency surpasses the one reported by Bednarek et al. of 9.2% in a COPD collective [7], but undercuts the prevalence of 22.3% observed in the Sleep Heart Health Study [8]. As compared to OSA alone, OS patients use to be older, present a more severe level of hypoxaemia and hypercapnia, but similar AHI and BMI [9]. These observations are largely consistent with our findings. Though AHI and BMI did not vary between OSA and OS patients, OS patients tended to be older without reaching statistical significance (*p* = 0.08). Capillary blood gas analysis revealed highest, though still normocapnic pCO2 levels amongst OS patients (*p* = 0.001). Capillary oxygen saturation did not differ between groups (*p* = 0.34). Nonetheless, capillary pO2 evidenced hypoxaemia in the OS cohort (65.4 ± 8.5 mmHg, *p* = 0.03 for intercohortal comparison).

Though myocardial stunning can cause transient sleep breathing disorders, leading to false positives [10], we did not observe differences in OSA or OS prevalence as a function of clinical cardiac presentation. Lowest left ventricular ejection fractions were measured in OS patients (non-significant). Ejection fraction negatively correlated with the AHI (Pearson´s *r* = -0.33, *p* = 0.04); the AHI, in turn, was related to N-terminal pro-brain natriuretic peptide (NT-proBNP, Pearson´s *r* = 0.82, *p* = 0.004) and inflammation markers (high-sensitivity C-reactive protein, hs-CRP: Pearson´s *r* = 0.63, *p* < 0.001; neutrophils: Pearson´s *r* = 0.39, *p* = 0.04). The same holds true for the oxygen desaturation index that correlated with NT-proBNP (Pearson´s *r* = 0.95, *p* < 0.001) and hs-CRP (Pearson´s *r* = 0.50, *p* = 0.009). While clearly, we cannot address the mechanisms underlying this suggestion, it supports the notion of OSA and OS as disorders accompanied by a pro-inflammatory systemic state that may not be confined to the airways, but may have systemic consequences.

The present study has several limitations including its single side character, the relatively small number of patients studied and the lack of additional longitudinal follow-up that would have permitted to evaluate the prognostic impact of OS on cardiac outcomes. Moreover, SDB screening was carried out by the two-channel SOMNOcheck micro^®^ device that relies on automatic data processing and might confer minor diagnostic accuracy than detailed polysomnography.

In conclusion, CAD seems to be accompanied by a substantial proportion of undiagnosed COPD and overlapping OSA whose occurrence does not vary over clinical cardiac presentation status.

## Data Availability

The datasets generated during and/or analyzed during the current study are available from the corresponding author on reasonable request.
